# Predictors of Business Return in New Orleans after Hurricane Katrina

**DOI:** 10.1371/journal.pone.0047935

**Published:** 2012-10-24

**Authors:** Nina S. N. Lam, Helbert Arenas, Kelley Pace, James LeSage, Richard Campanella

**Affiliations:** 1 Department of Environmental Sciences, Louisiana State University, Baton Rouge, Louisiana, United States of America; 2 Department of Geography & Anthropology, Louisiana State University, Baton Rouge, Louisiana, United States of America; 3 Department of Finance, Louisiana State University, Louisiana State University, Baton Rouge, Louisiana, United States of America; 4 Department of Finance and Economics, Texas State University, San Marcos, Texas, United States of America; 5 Tulane School of Architecture, Tulane University, New Orleans, Louisiana, United States of America; Tulane University School of Public Health and Tropical Medicine, United States of America

## Abstract

We analyzed the business reopening process in New Orleans after Hurricane Katrina, which hit the region on August 29, 2005, to better understand what the major predictors were and how their impacts changed through time. A telephone survey of businesses in New Orleans was conducted in October 2007, 26 months after Hurricane Katrina. The data were analyzed using a modified spatial probit regression model to evaluate the importance of each predictor variable through time. The results suggest that the two most important reopening predictors throughout all time periods were the flood depth at the business location and business size as represented by its wages in a logarithmic form. Flood depth was a significant negative predictor and had the largest marginal effects on the reopening probabilities. Smaller businesses had lower reopening probabilities than larger ones. However, the nonlinear response of business size to the reopening probability suggests that recovery aid would be most effective for smaller businesses than for larger ones. The spatial spillovers effect was a significant positive predictor but only for the first nine months. The findings show clearly that flood protection is the overarching issue for New Orleans. A flood protection plan that reduces the vulnerability and length of flooding would be the first and foremost step to mitigate the negative effects from climate-related hazards and enable speedy recovery. The findings cast doubt on the current coastal protection efforts and add to the current debate of whether coastal Louisiana will be sustainable or too costly to protect from further land loss and flooding given the threat of sea-level rise. Finally, a plan to help small businesses to return would also be an effective strategy for recovery, and the temporal window of opportunity that generates the greatest impacts would be the first 6∼9 months after the disaster.

## Introduction

It has been seven years since Hurricane Katrina hit the New Orleans region on August 29, 2005. The category-3 storm caused multiple levee breaches and inundated most of the city with floodwaters for more than a month. The death toll in the New Orleans region amounted to 1,600 lives, not including over 200 more deaths that occurred in nearby coastal Mississippi. Over half a million people evacuated, and except in a few high-ground areas, most of the businesses were closed. The recovery outlook was grim and uncertain. Although in the last few years, the greater New Orleans region is rebounding on some measures of economic growth such as increases in new businesses and wages, there are still troubling signs on other key economic, social, and environmental indicators such as low productivity and increase in social and economic disparities. The most current indicators show that greater New Orleans continues to recover and in some ways is performing “better than before” [1]. However, the latest census also confirms that New Orleans City (Orleans Parish) had 29 percent fewer residents in 2010 than in 2000 and the metropolitan area, which includes six more surrounding parishes (i.e. counties), had 11 percent fewer residents [1]. Moreover, the recovery pattern has been spatially uneven [2]. The Katrina disaster was undoubtedly one of the largest natural disasters that have impacted the United States; it is one that generated many questions to answer and lessons to learn. One key question remains: what induces businesses to return and recover from a major disaster? A better understanding of the factors that contribute to business owners’ decisions to close or open in a highly uncertain post-catastrophe environment, such as New Orleans after Hurricane Katrina, is needed to help develop effective policies for cities to respond to future disasters and mitigate their impacts. The society of southeastern Louisiana has become an unwitting test bed for trauma, uncertainty, and civic and business response in the past fifteen years. Since the late 1990s, the region has endured nine hurricanes, five large-scale evacuations, numerous tropical storms, an oil spill of historic proportions, two high-water events on the Mississippi River, the same Great Recession ongoing in the rest of the country, and a deluge of catastrophic proportions. This paper focuses on business’ response to Hurricane Katrina, but developments since the Katrina flood, some of which played central roles in the Hurricane Gustav (2008) and Hurricane Isaac (2012) experiences, are relevant to our findings and are addressed in the epilogue of this paper.

Studies directly addressing the relationships between business decisions and natural disasters remain sparse, and the findings from the sparse literature are often mixed and inconclusive [2]–[9]. Some studies argue that disasters have few effects beyond the immediate or short-term recovery periods [6], while others conclude that at most, natural disasters exacerbate existing trends [9], [10]. Yet another group of studies even suggest that climate-related disasters have long-term positive economic consequences [11], [12]. In general, three issues may contribute to the inconsistency of the findings from these studies. First, different studies may be based on data at different study scales. Conclusions based on studies at a broad regional scale, for instance, may conflict with those conducted at the metropolitan scale. Second, with a few exceptions [14], [15], existing studies have seldom incorporated the spatial information, such as business locations and their neighborhood attributes, and the spillover effect explicitly in their analysis. Third, studies on economic recovery after a major disaster often suffer from the lack of timely data, which are critical to accurate analysis and modeling. Modeling and prediction of how a city recovers obviously would require fine-scale analysis that considers the spatial effect, and such type of studies will need fine-scale empirical observations collected in a timely manner so that quantitative relationships can be measured and modeled.

We report in this paper the results of a probit regression analysis using the New Orleans business telephone survey data collected from a previous project [2]. The telephone survey data includes three rounds of surveys of about 1,400 business owners in Orleans Parish. The three timed surveys were conducted four months (December 2005), ten months (June 2006), and 26 months (October 2007) after Hurricane Katrina to track the responses of businesses and their opening statuses over a short term, intermediate, and longer time interval. In addition to the telephone survey data, detailed business reopening data were also gathered by repeated street surveys of businesses along three major avenues, resulting in a spatially complete subset of information for Orleans Parish business establishments. The changing patterns of business responses and their opening statuses in the Orleans Parish study area, as derived from the three telephone surveys, have been documented in [2], whereas the changing dynamics of business openings based on the street survey data have been analyzed in [14], [15]. In addition, another group recently reported their findings regarding the factors influencing business performance in New Orleans after Katrina [17], [18].

The purpose of this paper was to identify significant business opening predictors and their changing dynamics using the information derived from the third telephone survey. In this analysis, we incorporated the neighborhood information as well as a spatial variable in the probit model to evaluate the spatial spillover effect, which is how the opening of one business at one location in one time interval would impact the opening probabilities of its nearby businesses in the next time interval. The results from the probit regression analyses are a set of probabilities, showing how an increase or decrease in one variable would increase or decrease the probability of a business to open at different spaces and times. This information should provide insights into where, how, and when the aid would be most effective to speed economic recovery after a major disaster.

## Materials and Methods

### Data

The telephone survey data and some of the results have been described in detail in [2]. (Note: This research has been approved for an IRB exemption by the Louisiana State University Institutional Review Board (www.lsu.edu/irb). The exemption number is: E3552. The LSU IRB exemption includes a waiver of signed informed consent.)

In brief, three rounds of telephone surveys were conducted by calling all the businesses in Orleans Parish in three different periods: December 2005, June 2006, and October 2007. The purpose of multiple round surveys was to capture the spatial and temporal dynamics in business attitudes that influence their decision making and to enable subsequent quantitative modeling and prediction over both time and space. The listing of business establishments was derived from the August 2005 Louisiana Department of Labor Micro File for Economic Development in the greater New Orleans Area (which includes also two other parishes: Jefferson and St. Tammany). There were about 11,000 businesses in Orleans Parish before Hurricane Katrina. The file contains confidential information of about 45 variables for each establishment. Variables that were especially useful for this study included: the NAICS (North American Industry Classification System) code of the business, name of the businesses, physical address, telephone, contact person, longitude and latitude, ZIP code, census tract code, census block code, parish code, number of employees, and aggregate wages. The surveys were conducted with assistance from the Louisiana State University Public Policy Research Laboratory, and for the third survey, additional collaboration was from the Louisiana Recovery Authority.

To analyze the temporal dynamics of businesses’ opening probabilities using the spatial probit regression model, the dependent variable is the opening status of a set of businesses in Orleans Parish after Hurricane Katrina, with 0 and 1 representing closed and open, respectively. The information source was derived from one question in the third telephone survey, which was: “Did your business close following the 2005 Hurricanes? If yes, how long was it closed? (in months)”. Using the answers to this question it was possible to identify the exact location of the business, the census block group in which it was located, and its opening status at a certain time interval. These data were grouped into six time periods using a three-month interval to avoid the potential small-number problem. The time periods were: by September 2005 (T1), December 2005 (T2), March 2006 (T3), June 2006 (T4), and September 2006 (T5), and then for T6, it was from September 2006 to October 2007. We assumed businesses that had their telephones disconnected during the survey were still closed. Table 1 shows the number of newly opened businesses in each time interval.

**Table 1:** Number of businesses newly opened in each quarter.

	New openings	Cumulative openings	Remain closed
T0	173	173	1185
T1- Sep 05	268	441	917
T2- Dec 05	452	893	465
T3 - Mar 06	129	1022	336
T4- Jun 06	50	1072	286
T5- Sep 06	55	1127	231
T6- Oct 07	63	1190	168

Note: The first row shows the counts immediately after Katrina (August 29, 2005), which indicates that 173 businesses had never closed after Katrina. The total number of businesses in the sample was 1,358 (the sum of “cumulative openings” and “remained closed”). This number included those who completed the survey as well as those with disconnected phones.

Three types of predictors (independent variables) were included in the model for analysis. The first type of predictors refers to individual business attributes which include two variables: (1) Business size: we used the total wage of each business, obtained from the Department of Labor micro file, to indicate the size of the business. The variable was transformed into a natural logarithmic form for the analysis. (2) Disaster damage: we used flood depth at the business location as a proxy for disaster damage. The flood depth data (in meters) were obtained from the Katrina & Rita Clearing House Cooperative, managed by Louisiana State University.

The second type of predictors refers to the neighborhood characteristics surrounding the businesses. The neighborhood characteristics of a business, such as labor supply and customer purchasing power, are considered important factors influencing business’s decisions to reopen. The variables were chosen based on the business recovery and social vulnerability literature [6], [10], [11], [16]. A total of seven variables were selected, including population density, percent non-white population, percent female population, percent population with age under 18, percent population with age over 65, median household income (in natural logarithms), and percent renter occupied houses (Figures 2, 3, 4, 5, 6 in Information S1). These variables were obtained at the census block group level from the Census 2000. There are 485 census block groups in Orleans Parish. Ideally, updated social-demographic data at each time interval should be used in our probit models. However, such data are not available. We argue that using the same neighborhood statistics in our probit models throughout the 2-year period would help in providing insights into how the pre-Katrina neighborhood characteristics affect business’ decisions to return.

The third type of predictors refers to the spatial spillover effect. Classic statistical analysis considers each sample observation as independent, not being affected by other observations in the sample. In the case of business decisions, this seems unrealistic. The reopening decision of one business would certainly affect the decisions of its neighbors, hence incorporating a spatial effect variable is essential in modeling business’ decisions to return [14], [15]. In this study the spatial spillover effect was estimated based on the opening statuses of all the neighboring firms within a distance threshold, adjusted using an inverse distance-weighting method, and this leads to a variable with a range of values from 0–1. We considered only firms located within 1-km of the business of interest.

### Methods

To perform any spatial analysis it is necessary to include the business’ locations. Our dataset includes the address of each business. To transform the address into coordinates we used the Geocode tool of ArcGIS. The final result is a set of points, projected onto UTM coordinates (zone 15). Each point represents a business. Then, to associate the business locations with the census block group data, we performed a spatial join in ArcGIS (point-in-polygon operation). The flood depth information obtained for Orleans Parish was associated with the business locations using a point-in-raster operation in ArcGIS.

The spatial spillover effects were calculated using a Java program specifically designed for that task. Businesses in the sample were assigned a value of either 0 (closed) or 1 (open) based on their open/closed status. The Java program estimates the inverse-distance weighing value for each business based on the opening status of its neighbors within the 1-km threshold. The formula used to calculate the spatial variable *W_i_* is:
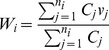
(1)and
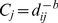
(2)where for each observation *i* we have a different number of neighbors *n_i_*, *V_j_* is the status of neighbor *j* (either 0 or 1), and *C_j_* is the inverse distance function value between observation i and its neighbor *j*, powered by *b*. Higher values of b would increase the importance of closer neighbors. We assumed that businesses observed the status of neighboring businesses in previous periods and used that observed status in making their own decisions to open or not open (i.e., we treated *V_j_* as a predetermined variable as opposed to treating it as a random variable). Based on Equation (1), the value of *W_i_* is affected not only by those neighbors who were open but also by those who were closed. *W_i_* is 1.0 when all the neighbors were open, and 0.0 when all of them were closed. In this study *b* was set to 1.0 (linear distance decay effect), which is considered reasonable since there was already a1-km proximity buffer imposed.

A probit regression model was used to model the probability of closed or open of a business given the nine explanatory variables and the spatial variable. The statistical package SAS was used to compute the model. A probit model is a form of generalized linear model. For an ordinary linear model, the equation is:
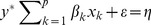
(3)where y* represents the value of the dependent variable (possibly latent), *x*’s are the explanatory variables, *P* is the number of predictor variables, *β*’s are the regression coefficients. For the probit model that examines a binary outcome *y*, based on an unobservable latent continuous y*, the equation is:
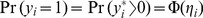
(4)where Φ is the standard normal cumulative distribution function. In this study, we added a spatial variable calculated at a previous time interval to represent the spatial spillover effect in the current time interval. Hence, the probit model employed in this study treats nearby business reopenings in the previous time interval as a predetermined variable:
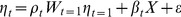
(5)where *W_t-1_* 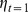 represents the spatial average of reopened businesses during the previous time interval, 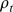 is the regression parameter associated with the spatial variable at time *t*, *X*’s are the explanatory variables and *β_t_,* the regression coefficients at time *t*.

The model used in this study is a simplified version of the one used in [15]. Both models examined the spatial effects (albeit on different data sets), but this study assumes that businesses condition on prior openings of other businesses while [15] treats openings of other firms during the same time interval as an explanatory variable.

## Results

**Figure 1:**
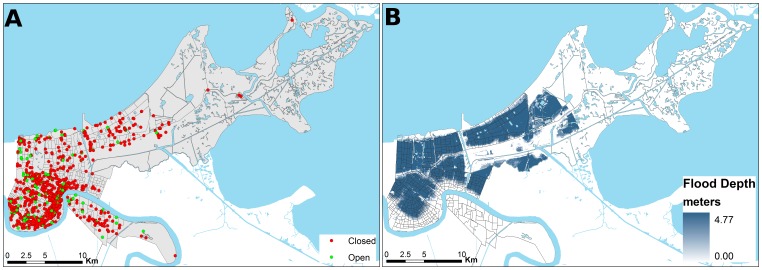
Businesses remained open immediately after Katrina. The total number of businesses in the sample was 1,358 in (A). Flood depth in New Orleans as of September 2, 2005 is shown in (B).

**Figure 2:**
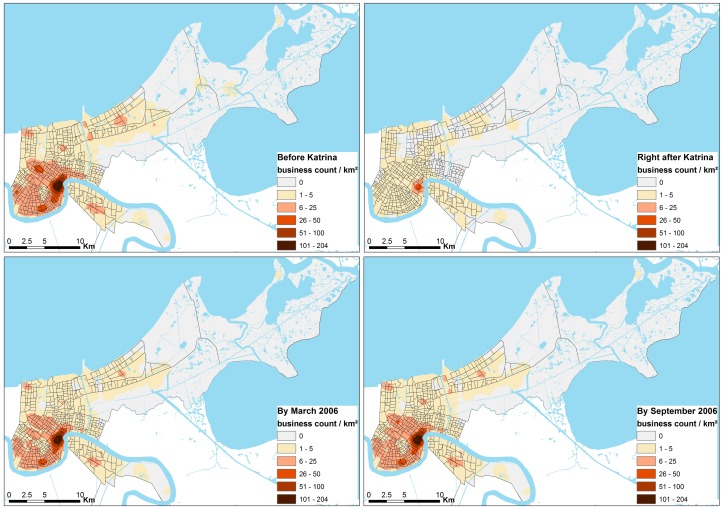
Kernel density maps of opened businesses in Orleans Parish in different time periods.

The six time periods analyzed were roughly in a three-month interval, except the last time period, T6, where the interval was 13 months (from October 2006 to October 2007) due to the smaller number of firms that remained closed one year after Hurricane Katrina. Table 1 shows that 168 businesses (12.4%) still remained closed at the time of the third survey, or 26 months after Katrina. Figure 1 shows the point locations of all the 1358 businesses in the sample, in reference to the 485 census block group boundaries. The flood depth as of September 5, 2005 is also shown. Figure 2 compares the generalized patterns of business reopenings before Katrina, immediately after Katrina, and six months and one year after Katrina using the kernel density smoothing method. The maps clearly show a clustered pattern of opened businesses in the early time periods, demonstrating a possible strong positive spatial effect. By the end of September 2005, most of the reopened (or never closed) businesses were located near the downtown natural levee area that had not been flooded. The trends and patterns of business reopenings through the six time periods are documented in Information S1.

### Probit Regression Results

The probit regression analysis was carried out to answer two related questions: what were the main predictor variables that could help explain the New Orleans business reopening process and how had the importance of the predictor variables changed over time? The criterion variable for the probit regression analysis is the business opening status, and the ten predictor variables included: business size, flood depth, the seven neighborhood variables before Katrina, and the spatial spillovers variable. We conducted the probit regression analysis for both cumulative and non-cumulative scenarios. In the cumulative scenario, all 1358 businesses were included in the analysis at each time interval, whereas in the non-cumulative scenario, businesses that had already opened in previous time intervals were excluded from the analysis. The cumulative scenario reflects the overall patterns and trends as a snapshot, whereas the non-cumulative scenario pinpoints the factors that are associated with the new openings at specific time intervals. Tables 2 & 3 lists the regression coefficients (*B*) and marginal effects (*Meff*) of each variable through time. The numbers of open and closed businesses (*n1, n0*) in each time interval for both scenarios were also listed. The probit models derived for each time period generally had good fits, with their respective likelihood ratios significant at *p*<0.05 levels for all time periods in the cumulative scenario and except for Times 4 and 6 in the non-cumulative scenario.

In the cumulative scenario (Table 2), wages and flood depth were found to be statistically significant throughout the six time periods. Larger businesses had higher reopening probabilities (positive *B* coefficients), whereas businesses that had greater flood depth had lower reopening probabilities (negative *B* coefficients). Surprisingly, with only one exception, none of the pre-Katrina neighborhood variables were found to be significant throughout the time periods. During the last time period, the variable percent population under 18 years old became a significant positive predictor, though its marginal effects were small (0.003). The spatial spillover variable was a significant positive predictor for the first four time periods. After that, the variable became insignificant.

The non-cumulative scenario exhibited a more varying trend, which is expected, because the models included only the businesses that had newly reopened in the previous period (Table 3). Business size (wages) was identified as a significant positive predictor only in the first two periods, whereas flood depth was a significant negative predictor only in the first three periods. Similar to the cumulative scenario, the pre-Katrina neighborhood attributes did not seem to stand out as significant predictors consistently over time except in four cases: percent non-white population was a significant negative predictor in Time 2, percent population under 18 years a negative predictor in Time 3, percent renter-occupied houses a negative predictor in Time 4, and percent female population a positive predictor in Time 5. The spatial spillover variable was found to be a significant positive predictor only in the first period (September 2005, one month after Katrina); it became insignificant in Time 2 to Time 4, and then reversed into a significant negative predictor in Time 5 (September 2006, a year after Katrina).

A major advantage of using the probit regression model is that the marginal effects derived from the models can be used to evaluate how an increase (or decrease) in value of a variable affects the reopening probability of an establishment (*Meff* in Tables 2 & 3). We explain below the results from the cumulative scenario. During the first time period, an increase in one unit of the natural logarithm of the wages would increase the opening probabilities by 0.010 (1.0%), while in the second time period it would increase the opening probabilities by 0.012 (1.2%). The marginal effects of this variable were very consistent throughout the six time periods. It is noted that since the wage variable was used in a natural logarithm form, the marginal effects will be non-linear. For instance a change of one unit for a business with *ln*(wages) = 1 to a new value 2 would mean a change of $4.67, while an increase from *ln*(wages) = 10 to a new value 11 would mean a change of $37,847.68. Thus the effects of increasing business size to increase the reopening probabilities are larger in the small and medium categories than in the large-size category.

**Table 2:** Probit regression results based on cumulative cases.

Cumulative	T1-Sep05		T2-Dec05		T3-Mar06		T4-Jun06		T5-Sep06		T6-Oct07	
	B	Meff	B	Meff	B	Meff	B	Meff	B	Meff	B	Meff
ln(wages)	0.028	0.010	0.034	0.012	0.042	0.012	0.046	0.012	0.046	0.011	0.046	0.009
flood_dh	−0.155	−0.055	−0.407	−0.142	−0.378	−0.111	−0.400	−0.108	−0.321	−0.078	−0.289	−0.057
Pop_dens	0.000	0.000	0.002	0.001	0.003	0.001	0.002	0.001	0.002	0.000	0.001	0.000
p_nwhite	−0.001	0.000	−0.003	−0.001	−0.002	−0.001	−0.002	0.000	−0.002	0.000	−0.003	−0.001
p_female	0.000	0.000	0.004	0.002	0.005	0.001	0.003	0.001	−0.001	0.000	−0.008	−0.002
p_pop<18	0.003	0.001	0.008	0.003	0.001	0.000	0.002	0.000	0.006	0.001	0.014	0.003
p_pop>65	−0.001	0.000	0.000	0.000	−0.007	−0.002	−0.006	−0.002	−0.007	−0.002	−0.004	−0.001
ln(mhhi)	−0.068	−0.024	−0.104	−0.036	−0.190	−0.056	−0.167	−0.045	−0.183	−0.044	−0.135	−0.026
p_renters	−0.001	0.000	0.000	0.000	0.001	0.000	0.001	0.000	−0.001	0.000	0.001	0.000
spatial	0.617	0.219	0.574	0.200	0.757	0.223	0.589	0.159	0.369	0.089	0.190	0.037
intercept	0.014		0.874		1.737		1.658		2.403		2.337	
likelihood *R*	19.851		80.431		98.701		86.278		52.070		37.701	
n1/n0	441	917	893	465	1022	336	1072	286	1127	231	1190	168

Note: *B* denotes regression coefficients and *Meff* marginal effects. Coefficients and likelihood ratio with *p*<0.05 are highlighted. n1, n0 are numbers of businesses opened and closed used in each time period.

**Table 3:** Probit regression results based on non-cumulative cases.

Non-Cumulative	T1-Sep05		T2-Dec05		T3-Mar06		T4-Jun06		T5-Sep06		T6-Oct07	
	B	Meff	B	Meff	B	Meff	B	Meff	B	Meff	B	Meff
ln(wages)	0.046	0.014	0.049	0.018	0.016	0.005	0.049	0.011	0.041	0.009	0.017	0.005
flood_dh	−0.155	−0.046	−0.375	−0.139	−0.249	−0.083	−0.198	−0.045	0.110	0.025	−0.003	−0.001
Pop_dens	0.001	0.000	0.002	0.001	0.002	0.001	−0.005	−0.001	0.002	0.000	0.008	0.003
p_nwhite	0.001	0.000	−0.009	−0.003	0.006	0.002	−0.002	0.000	0.000	0.000	0.006	0.002
p_female	0.006	0.002	0.006	0.002	0.010	0.003	−0.023	−0.005	0.064	0.014	−0.029	−0.009
p_pop<18	−0.007	−0.002	0.001	0.001	−0.023	−0.008	0.014	0.003	−0.021	−0.005	−0.002	−0.001
p_pop>65	−0.001	0.000	0.004	0.002	0.009	0.003	0.000	0.000	−0.012	−0.003	−0.007	−0.002
ln(mhhi)	0.111	0.033	−0.120	−0.044	0.067	0.022	−0.429	−0.097	0.073	0.016	0.206	0.064
p_renters	0.005	0.001	−0.001	0.000	0.004	0.001	−0.012	−0.003	−0.010	−0.002	−0.003	−0.001
spatial	0.510	0.151	0.266	0.099	0.345	0.115	−0.129	−0.029	−0.876	−0.197	−0.627	−0.196
intercept	−2.851		0.851		−2.242		4.727		−3.669		−1.209	
likelihood *R*	24.096		86.785		25.440		13.388		29.331		8.866	
n1/n0	268	917	452	465	129	336	50	286	55	231	63	168

Note: Same notations as in Table 2.

The flood depth variable had the most consistent marginal effects among all the predictor variables on the reopening probabilities. In the first time period, an increase in one meter of flood depth would decrease the opening probability by −0.055 (−5.5%). The marginal effects of flood depth peaked to −0.142 (14.2%) in the second period, and the effects continued to dominate for the entire 26 months. In the last time period, the flood depth effects returned to about the same magnitude as the first period (−0.057or 5.7%).

As already mentioned above, the effects of the pre-Katrina neighborhood variables were not significant except in one case. Even for the lone case, the marginal effects were small: in Time 6, a one percent increase in population under 18 would increase the reopening probabilities by 0.003 (0.3%).

The spatial variable has a range of values between 0 and 1, with 1 representing the scenario where all the surrounding businesses (within 1 km) were open and 0 where all were closed. In the first time period, an increase in 0.1 unit of this variable would increase the opening probabilities by 0.0219 (2.19%). As time progressed, the positive effects of the spatial variable diminished and became insignificant one year after the disaster. The impacts of the spatial variable were by far the largest among all the variables, but only for the first four time periods.

## Discussion

To a large extent the results from this study support findings reported from past literature. However, there are also contradictions. We note that comparing results among different studies would be problematic, as different studies may have different definitions of variables, time scales, analytical methods, and/or types of disasters. Also, the findings from the sparse literature on business decisions and natural disasters are often mixed and inconclusive. Here, we compare our findings only with those that are most directly related to the business recovery in New Orleans after Katrina [14], [15], [17], [18]. Corey and Deitch studied the factors affecting business performance immediately after Hurricane Katrina in New Orleans by surveying 183 surviving businesses 6∼8 months after Katrina [17]. Based on the responses of the survey they concluded that storm damage and post-disaster problems were significant negative predictors on business performance (e.g., better, same, or worse than before Katrina). Also, loss of customers and workers due to population relocation had the greatest impact on the firm’s performance. These are findings similar to the business survey responses documented in [2], and they are also supported by this study. However, Corey and Deitch also reported that business size was not related to the firm’s performance [17]. This latter result is not comparable to the findings in this study, since we focus on reopening probabilities rather than performance after opening. The same research group also conducted another similar study, which tracked the long-term business performance four years after Katrina based on responses from another survey of 186 surviving businesses [18]. Only three variables were found significant in explaining firm’s performance. These included two variables related to population issues (loss of customer base and labor shortage) and a macro variable on “lack of federal assistance”; all were negative predictors. While these two studies provide useful perspectives on business performance after a disaster, the lack of spatial information associated with the businesses in the sample (e.g., location and neighborhood attributes), the small number of observations used relative to the large heterogeneous study area (four parishes), and the survival bias could contribute to some differences in findings between their studies and the current one.

LeSage and others examined business reopening decisions after Hurricane Katrina in New Orleans using a data set that was collected under the same project as the current one [2]. The data set included 673 establishments along three major thoroughfares in New Orleans [14]. The researchers applied a spatial auto-regressive (SAR) variant of the conventional probit regression model that was developed previously in [13]. The SAR probit model allows for interdependence between firms’ decisions on openings (spatial spillovers), and the model estimates can be used to evaluate the direct and indirect (spatial spillovers) effects of each variable on the reopening probabilities. LeSage and others used the opening status of 11 nearest neighbors (after a number of tests) to calculate the spatial variable in the SAR probit model. Their results indicated that flood depth was a consistent significant negative predictor on the reopening probabilities throughout the three time periods, a result that is also confirmed by this study. Low socio-economic status of customers, relative to middle status customers, also had a consistent significant negative impact for all three periods. Small firms, relative to medium sized, had a negative influence, though this variable was significant only in the first period. Sole proprietorship had a positive influence, though the variable was significant only in the first two periods. The only neighborhood-level variable tested, (logged) median household income, exhibited a positive effect, but the variable was insignificant throughout the periods. This latter finding is also confirmed by this study. Last but not least, the spatial spillovers effect was a significant positive predictor on the reopening probabilities of the firms for at least one year, even though the derivation of the spatial variable is different between the two studies. In general, the findings from LeSage and others [14] and the current one are similar in that both studies found that effects of the explanatory variables diminished as time progressed and that significant spatial interdependence in business decisions existed, especially in the early stages post Katrina. Flood depth was found to be the most pervasive, consistently negative factor in both studies. Similarly, the lack of low economic status customers (and hence labor shortage), a problem related to low population return in certain segments of population in New Orleans, was contributing negatively to the reopening probabilities. In addition, both studies show that neighborhood’s median household income was not a significant factor influencing business’ decisions to reopen.

### Conclusions

This study addresses the effects of both businesses’ own attributes and their surrounding spatial characteristics on business reopening probabilities. The incorporation of the spatial attributes has provided useful insights into the business recovery processes after a major catastrophe. We can summarize our findings and their implications as follows:

- First, our results clearly indicate that smaller businesses had lower reopening probabilities than larger ones, and their marginal effects were fairly consistent throughout the six time periods. This result agrees with those derived from past studies conducted elsewhere [4]–[10], [19], as well as in part with [14]. More importantly, this study shows that the marginal effects of business size are non-linear, such that the effects of increasing business size on its reopening probabilities would be larger for smaller businesses than for larger businesses. A closer look at the non-cumulative scenario shows that the business size variable stopped being significant by March 2006, approximately 6∼9 months after Katrina. These results suggest that small businesses had the greatest difficulty reopening, and policies designed to help business recovery should consider the temporal dimension. In this case, the window of opportunities was within 6∼9 months after the disaster. Furthermore, the help would be most effective for smaller businesses than for larger ones.
- Second, business decisions regarding reopening are spatially interdependent, and are closely linked with the population return in their communities. A major disaster causes massive population displacements, and in many cases the population remains mobile for several months. After a disaster, businesses are affected by the reduced number of customers due to their displacements, reduced purchasing power of their clients, and reduced supply of labor. In the case of New Orleans, population displacement is difficult to study at the micro level due to the lack of up-to-date population return data [1]. The 2006 American Community Survey (ACS) conducted by the U.S. Census Bureau showed that the return of young people and families with children to New Orleans was lower compared to other segments of the population, while the returning population was disproportionally composed of white, childless and better educated population [20]. Our results reflect that reopening probabilities were somewhat impacted by the population return, although the marginal effects were not strong. We argue that the effects of neighborhood socioeconomic attributes might have been masked by the effects of flood depth and the spatial spillovers. Overall, the implication of this second finding is that policies designed to improve the business return should also consider spatial spillovers and resident return. “Do what your neighbors do” after a disaster seems to be a critical decision making factor for business’ decision to reopen, especially during the first 9 months after the disaster [15].
- Perhaps the most devastating characteristic regarding the long-term economic recovery in New Orleans after Hurricane Katrina is its vulnerability to large-scale flooding. The flood depth or flood damage variable has been repeatedly identified as the most persistent and dominant factor by several studies including the current one. Businesses located in higher flood-depth areas had lower probabilities of reopening, and the marginal effects of this variable were large relative to other model variables. This indeed underscores the importance of flood reduction measures in New Orleans needed to increase resiliency and maintain long-term sustainability. The geometric shape of the flooding pattern also matters in terms of the speed of recovery that could ultimately affect the overall rate of recovery. It is reasonable to postulate that a compact-shaped disaster area is more difficult to access and would take longer to recover than an elongated-shaped impacted area (e.g., flooding along a river or coastline or damage along a tornado path), and that should apply to all types of disasters. In New Orleans, the flooded area was large and compact like a bowl, making access to the disaster area more difficult and the recovery process much longer. Access to major infrastructures such as electricity, telecommunication, roads, and public transportation were delayed due to large-scale flooding. The associated impacts or collateral damages were high, affecting even businesses that were not flooded because of the spatial interdependence among businesses themselves as well as their communities. Lam and others previously argued for an adequate plan to protect the infrastructure and mitigate disaster impacts, and suggested that policy designed to reduce vulnerability and boost economic recovery should be applied in a timely manner before business decisions to return or close are made [2]. This study confirms the findings in [2] through the use of a probabilistic model. Furthermore, the findings here imply that the plan should include a spatial design that would protect certain critical structures or access points (e.g., points where telecommunication, electricity, transportation are protected during the disaster strikes) to keep certain businesses open to generate more and quicker positive spatial spillover effects. Such a design would more likely reduce the impacts and speed the recovery.

So, what is the main message from this study? It is clear that prolonged flooding is a key negative factor affecting the long-term recovery of the region. Partly as a result of this, since 2006, the U.S. Army Corps of Engineers has installed gates on outfall canals, closed a major navigation canal, erected a Dutch-style surge barrier, raised levee heights, installed pumps, and strengthened floodwalls. Current estimates are that metro New Orleans is protected from storms that have a one-percent chance of occurring in any given year. While that does not include a Katrina-like storm, the new system does significantly reduce the risk that businesses might suffer the sort of disruption that occurred in 2005, thus increasing confidence of potential investors from outside the area. In fact, Hurricane Isaac, which struck exactly seven years to the day after Katrina, showed that the flood control measures had materially helped the areas that they protected, as much of the damage occurred outside of these structures.

Nonetheless, the debate continues on whether coastal Louisiana will be sustainable or too costly to protect from further land loss and flooding given the long-run threat of sea-level rise and global warming. In the long-run, a much more robust, long-term effort to reduce flood risk would be necessary, which would likely be expensive and involve competition among the needs from different sectors (for example, a pipeline crisscrossing sensitive wetlands could speed the land loss process). Given the threat of sea-level rise and subsidence in the region, as well as human extraction of resources (e.g., pipelines and canals), a dynamical system approach to studying the interactions between both natural and human elements would be most beneficial to the economic health and sustainability in the region.

As for the near term, our study has shown once again that plans to reduce flooding risks and its collateral damage are essential for recovery, and these plans would be most effective when carried out in the first 6∼9 months after a major disaster, which is the window of opportunity for building resilience and healthy recovery.

### Epilogue

Three years after Katrina, Hurricane Gustav (September 1, 2008) threatened New Orleans and instigated a mandatory evacuation. The levees held and no flooding occurred, but the interruption to all forms of social and economic activity evoked the question of whether investors and businesses could tolerate the annual possibly of multiple full-scale evacuations even without disastrous consequences. Exactly seven years after Katrina, Hurricane Isaac (August 29, 2012) approached the city on a track that positioned the metropolis in the dreaded northeastern quadrant. But because the Army Corps of Engineers had by then completed the Risk Reduction System, authorities decided against issuing a mandatory evacuation. The storm proved to be more damaging than expected, and well over a million people suffered three to six days without electricity. Nevertheless, the decision against evacuation spared citizens, schools, and businesses a costly and potentially dangerous week-long journey of lost work time, and proved to be a sound one. The seven years of recovery and progress since Hurricane Katrina, and the experiences of Gustav and Isaac, have introduced new variables into the formula of business investment and return to an urban economy buffeted by repeated trauma.

## Supporting Information

Information S1 (DOCX)
