# Supplementary material for: Predictors of Business Return in New Orleans after Hurricane Katrina
Source: PLoS One. 2012 Oct 24;7(10):e47935. doi: 10.1371/journal.pone.0047935 (PMC3480509; doi:10.1371/journal.pone.0047935)
Supplement: Information S1 — (DOCX) [file pone.0047935.s001.docx]

**Supporting Information S1**

**Trends and Patterns of Reopenings**

The six time periods analyzed were roughly in a three-month interval, except the last time period, T6, where the interval was 13 months (from October 2006 to October 2007) due to the smaller number of firms that remained closed one year after Hurricane Katrina (Table 1; Figure S1). Of the 1358 businesses included in this study, 173 had never closed after Katrina. By September 2005, one month after Katrina, 268 businesses had reopened, and by December 2005, 452 new openings were added. The cumulative total number of openings four months after Katrina (by December 2005) was 893, about 65.8% of all businesses in the sample. However, it is noted that this percentage may not represent the real business reopening rate, because the large number of businesses that was in the “no answer” category was not counted as “closed” in this study. Hence, the reopening rate tabulated in this study is a much higher, more optimistic estimate than the one (about 25% by December 2005) reported in [2]. The latter study counted businesses that did not answer the phone after five attempts as “closed”. Table 1 also shows that 168 businesses (12.4%) still remained closed at the time of the third survey, which was conducted in October 2007 or 26 months after Katrina.

A comparison between opened and closed businesses in terms of their own attributes (wages as a proxy of business size and flood depth as a proxy of flood damage) and their neighborhood attributes at the census block group level through time reveals some interesting patterns. In the cumulative scenario (Table S1), businesses that had never closed or had reopened within a month after Hurricane Katrina generally had higher wages (in natural logarithms) and lower flood depth (in meters). The opened businesses were located in census block groups that by and large had lower population density and lower percentages of non-white population, female population, and population below 18 years old. Somewhat surprising is that there were no observable differences between the opened and the closed businesses in terms of the block group’s medium household income and percent renter occupied houses.

As expected, statistics tabulated in the non-cumulative scenario were not as stable as that of the cumulative scenario, especially for the last two time intervals. By September 2006 (Time 5), a year after Katrina, the effects of most variables on the opening probabilities seemed to have reversed. Unlike previous time periods, businesses opened in this time period were found to have higher flood depth, and they were located in communities (block groups) that had higher percentages of non-white population, female population, and population below 18 years old. This finding can be interpreted by the fact that factors that were critical (e.g., flood depth) in the early stages were not as critical one year after Katrina. Neighborhoods that were not conducive to openings at the beginnings might now have gradually recovered after one year and became more attractive to new openings. It is noted that business size continued to make a difference throughout all time periods in both cumulative and non-cumulative scenarios, where opened businesses consistently had higher wages than closed businesses.

**Table S1.** Comparison of the average neighborhood attributes between businesses closed and opened at different time periods using cumulative (top table) versus non-cumulative (bottom table) cases. *

| Cumulative | All_BG | T1-Sep05 | | T2-Dec05 | | T3-Mar06 | | T4-Jun06 | | T5-Sep06 | | T6-Oct 07 | |
| --- | --- | --- | --- | --- | --- | --- | --- | --- | --- | --- | --- | --- | --- |
|  |  | open | closed | open | closed | open | closed | open | closed | open | closed | open | closed |
| ln(wages) | 9.7 | 10.0 | 9.6 | 9.9 | 9.4 | 9.9 | 9.4 | 9.9 | 9.3 | 9.9 | 9.2 | 9.8 | 9.1 |
| flood_dh | 0.4 | 0.3 | 0.4 | 0.3 | 0.6 | 0.3 | 0.6 | 0.3 | 0.6 | 0.4 | 0.6 | 0.4 | 0.6 |
| pop_dens | 25.0 | 23.6 | 25.6 | 24.6 | 25.6 | 24.7 | 25.8 | 24.6 | 26.3 | 24.7 | 26.1 | 24.9 | 25.1 |
| p_nwhite | 46.2 | 43.7 | 47.3 | 41.8 | 54.5 | 43.0 | 55.8 | 43.7 | 55.4 | 44.5 | 54.1 | 45.2 | 52.7 |
| p_female | 48.0 | 47.7 | 48.2 | 47.5 | 49.1 | 47.6 | 49.4 | 47.6 | 49.7 | 47.9 | 48.8 | 47.9 | 48.9 |
| p_pop<18 | 16.5 | 15.1 | 17.1 | 14.8 | 19.6 | 15.0 | 20.8 | 15.3 | 20.8 | 15.7 | 20.3 | 16.0 | 19.8 |
| p_pop>65 | 13.2 | 13.1 | 13.3 | 13.4 | 12.8 | 13.5 | 12.4 | 13.4 | 12.4 | 13.4 | 12.3 | 13.3 | 12.5 |
| ln(mhhi) | 10.1 | 10.2 | 10.1 | 10.2 | 10.1 | 10.2 | 10.1 | 10.2 | 10.1 | 10.2 | 10.1 | 10.2 | 10.1 |
| p_renter | 53.6 | 54.2 | 53.3 | 53.7 | 53.5 | 54.0 | 52.4 | 53.9 | 52.5 | 53.6 | 53.7 | 53.6 | 53.8 |
| count | 1358 | 441 | 917 | 893 | 465 | 1022 | 336 | 1072 | 286 | 1127 | 231 | 1190 | 168 |

| Non-cumulative | All_BG | T1-Sep05 | | T2-Dec05 | | T3-Mar06 | | T4-Jun06 | | T5-Sep06 | | T6-Oct 07 | |
| --- | --- | --- | --- | --- | --- | --- | --- | --- | --- | --- | --- | --- | --- |
|  |  | open | closed | open | closed | open | closed | open | closed | open | closed | open | closed |
| ln(wages) | 9.7 | 10.2 | 9.6 | 9.8 | 9.4 | 9.6 | 9.4 | 9.9 | 9.3 | 9.6 | 9.2 | 9.5 | 9.1 |
| flood_dh | 0.4 | 0.3 | 0.4 | 0.3 | 0.6 | 0.4 | 0.6 | 0.5 | 0.6 | 0.8 | 0.6 | 0.7 | 0.6 |
| pop_dens | 25.0 | 25.0 | 25.6 | 25.6 | 25.6 | 25.1 | 25.8 | 23.0 | 26.3 | 27.2 | 26.1 | 28.8 | 25.1 |
| p_nwhite | 46.2 | 44.9 | 47.3 | 39.9 | 54.5 | 51.2 | 55.8 | 57.9 | 55.4 | 60.8 | 54.1 | 58.0 | 52.7 |
| p_female | 48.0 | 47.5 | 48.2 | 47.2 | 49.1 | 48.2 | 49.4 | 47.9 | 49.7 | 53.4 | 48.8 | 48.3 | 48.9 |
| p_pop<18 | 16.5 | 15.1 | 17.1 | 14.6 | 19.6 | 16.4 | 20.8 | 20.8 | 20.8 | 23.3 | 20.3 | 21.4 | 19.8 |
| p_pop>65 | 13.2 | 13.1 | 13.3 | 13.7 | 12.8 | 13.8 | 12.4 | 12.4 | 12.4 | 12.9 | 12.3 | 11.8 | 12.5 |
| ln(mhhi) | 10.1 | 10.1 | 10.1 | 10.2 | 10.1 | 10.1 | 10.1 | 10.0 | 10.1 | 10.2 | 10.1 | 10.1 | 10.1 |
| p_renter | 53.6 | 55.6 | 53.3 | 53.2 | 53.5 | 56.3 | 52.4 | 51.9 | 52.5 | 47.4 | 53.7 | 53.4 | 53.8 |
| count | 1358 | 268 | 917 | 452 | 465 | 129 | 336 | 50 | 286 | 55 | 231 | 63 | 168 |

* The column “All_BG” contains the average of all census block groups in Orleans Parish. Except for “wages” and “flood_depth” where their averages were calculated from individual firms’ attributes, all other variables were calculated based on the statistics of the census block groups in which the firms were located.


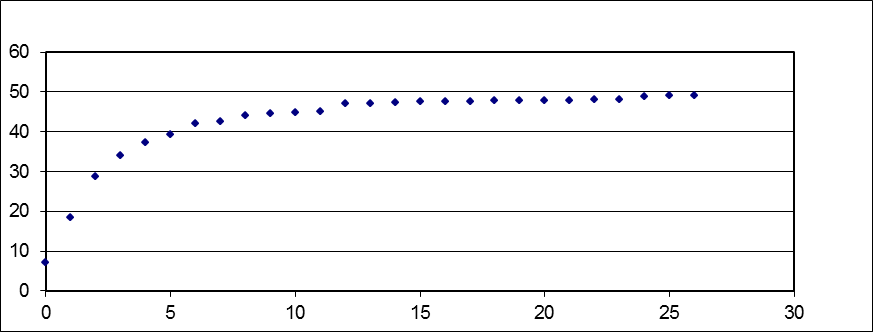


Time

(Month)

% Businesses

**Figure S1**. Cumulative business return rate based on data from the third survey plotted in a monthly interval.


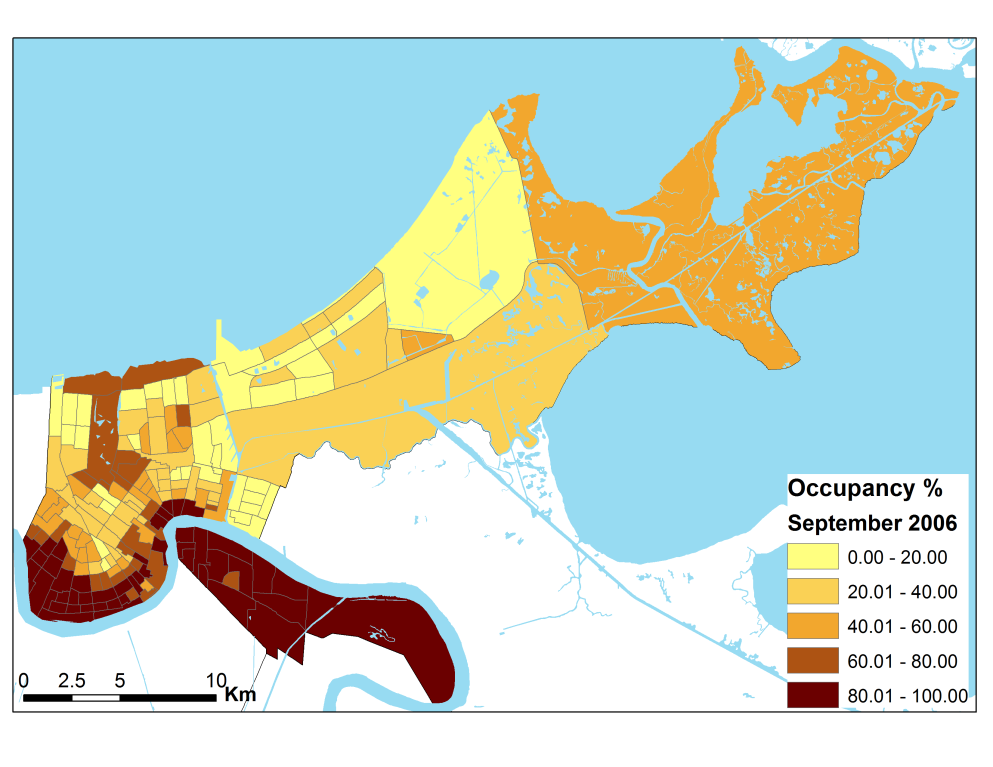


**Figure S2**. Percentage of address actively receiving mail by September 2006 at the census tract level, using U.S. Postal Service data.


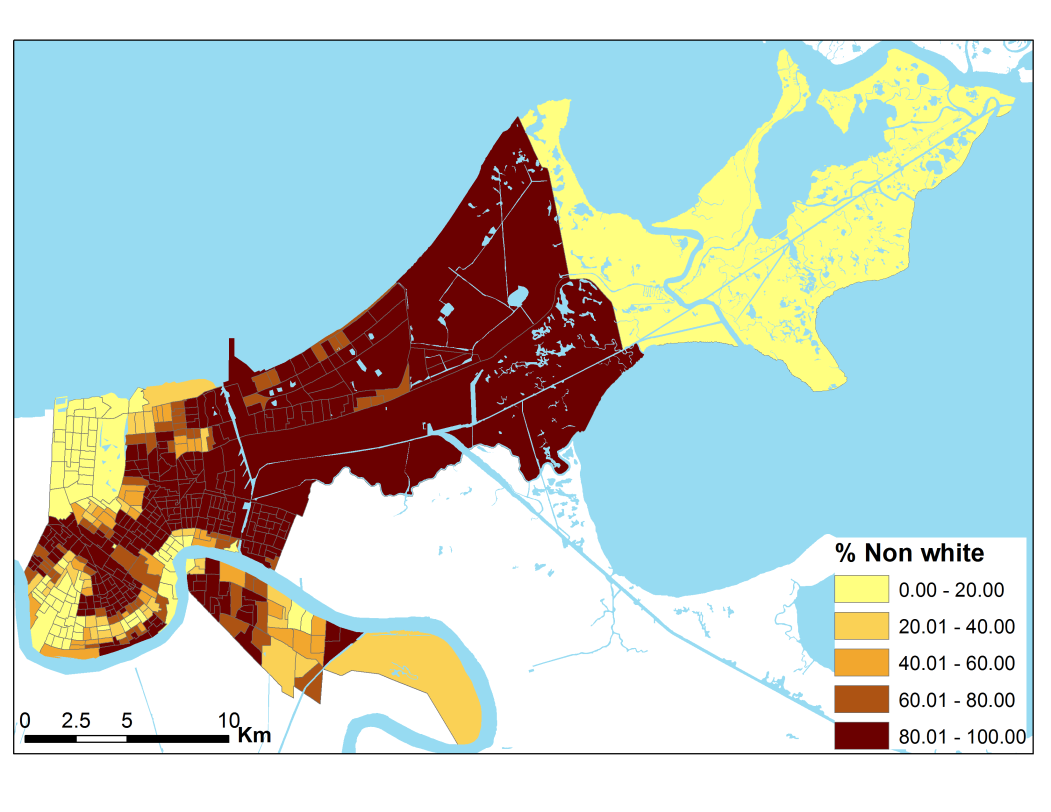


**Figure S3**. Percentage of Non white population at the census block group level using census 2000 data.


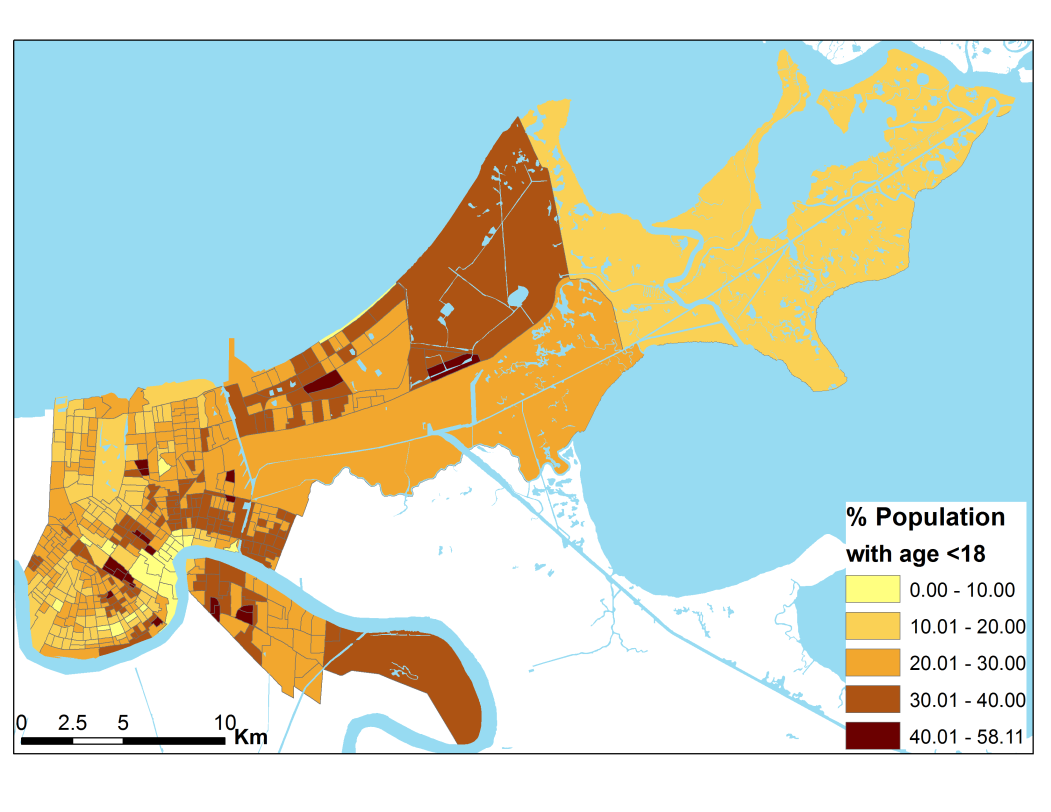


**Figure S4**. Percent of population under 18 years old at the census block group level using census 2000 data.


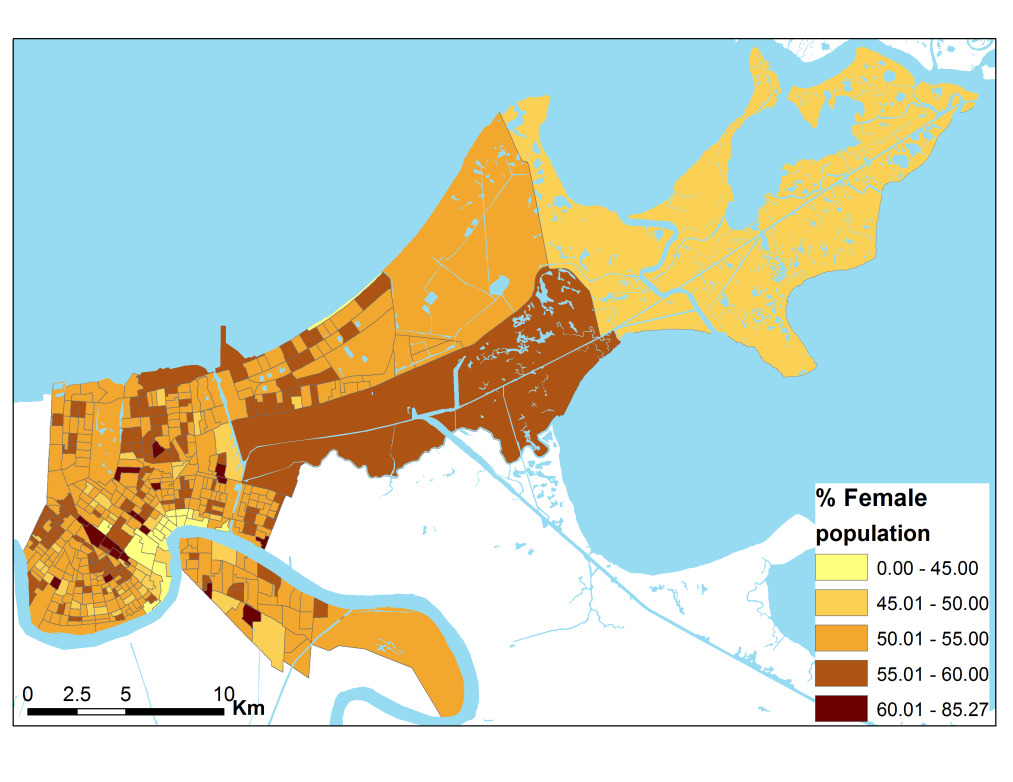


**Figure S5**. Percent of female population at the census block group level using census 2000 data.


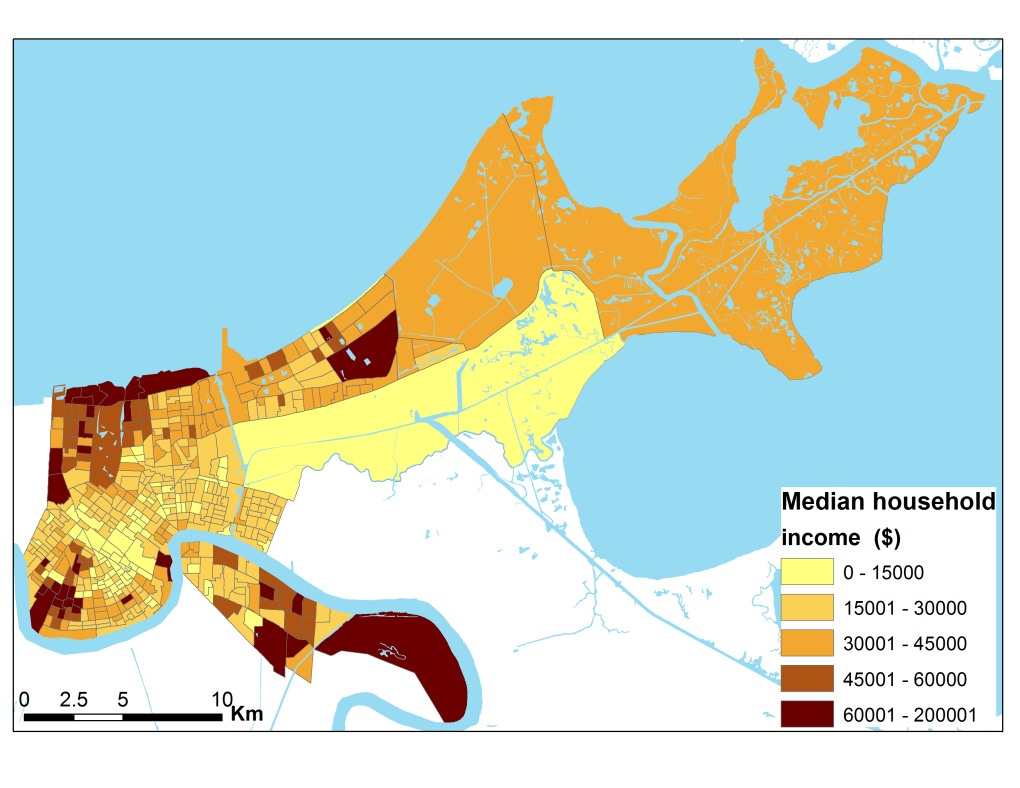


**Figure S6**. Median household income at the census block group level using census 2000 data.
